# MATERIAL DEPRIVATION AND LONELINESS AMONG OLDER ADULTS IN HONG KONG

**DOI:** 10.1093/geroni/igad104.0071

**Published:** 2023-12-21

**Authors:** Vivien Foong Yee Tang, Kee Lee Chou

**Affiliations:** The Education University of Hong Kong, Hong Kong, Hong Kong; The Education University of Hong Kong, Hong Kong, Hong Kong

## Abstract

Given that poverty as a risk factor on loneliness is understudied, this study examined the relationship between material deprivation and loneliness of older adults in Hong Kong, the mediation effects of social support and social network, and the moderation effects of social support and social engagement. A two-wave data were collected from 1,696 Chinese older adults between 2015 (T1) and 2017 (T2). All Chinese older adults aged 60 years and above, with 53.9% female. Older adults were asked about their level of loneliness, how material deprived they were, social support, social network, social engagement, demographic information, and self-rated health status. The result indicated that older adults who are more materially deprived reported a significantly higher level of loneliness at T2 when controlling for demographic variables and loneliness at baseline. Furthermore, we found that social support and the number of close children were full mediator. A significant moderator was found in neighbourhood collective efficacy and community participation specifically engagement in cultural activities. Hence, our results suggest the need to strengthen the anti-poverty policy measures for materially deprived, develop interventions focusing on improving social support and social network and enhance existing social engagement programmes that promote community participation and neighbour collective efficacy for older adults in Hong Kong.

